# Clinicopathologic Findings in Mass Forming ANCA-Associated Vasculitis

**DOI:** 10.1016/j.ekir.2022.09.019

**Published:** 2022-09-29

**Authors:** Sarwat I. Gilani, Mariam P. Alexander, Samih H. Nasr, Mary E. Fidler, Naoki Takahashi, Lynn D. Cornell

**Affiliations:** 1 Department of Laboratory Medicine and Pathology, Mayo Clinic, Rochester, Minnesota, USA; 2 Department of Radiology, Mayo Clinic, Rochester, Minnesota, USA

**Keywords:** antineutrophil cytoplasmic antibody, crescentic glomerulonephritis, granulomatous inflammation, IgG4, interstitial nephritis

## Introduction

Antineutrophil cytoplasmic antibody (ANCA)-associated vasculitis (AAV) is a systemic inflammatory disease predominantly affecting small blood vessels, with few or no immune deposits, associated with myeloperoxidase ANCA or proteinase 3 ANCA.^1^ AAV typically presents with constitutional symptoms, involvement of upper and/or lower respiratory tract, and kidneys.^2^, ^3^, ^4^ AAV can form inflammatory masses in various organs, including the orbit, kidney, and even pancreas, similar to IgG4-related disease (IgG4-RD).^5^, ^6^, ^7^, ^8^, ^9^^,^S1 Renal masses due to ANCA-associated disease are rare or under-recognized, and to our knowledge only published as case reports.S2

Renal involvement by AAV usually manifests as renal failure, hematuria, and/or proteinuria. On renal biopsy, AAV typically reveals necrotizing and crescentic glomerulonephritis. A subset of AAV cases can show increased IgG4+ plasma cells in the kidney and in other organs.S3,S4 Indeed, some authors have (perhaps erroneously) proposed an “overlap syndrome” of AAV and IgG4-RD.S5 These 2 entities can be confused, and their overlapping features can lead to misdiagnosis and inadequate treatment. Therefore, we were interested in investigating clinical and histological characteristics in a series of renal mass-forming AAV, and in particular how it relates to one entity it can mimic, IgG4-RD.

## Results

### Clinical Characteristics

The patients’ clinical characteristics are shown in Table 1. The cohort consists of 10 adults (7 men and 3 women) with an average age of 67 years (range: 55–78 years). Half (5 of 10) of the patients presented with fever, fatigue, and/or night sweats. Mean serum creatinine at presentation was 2.92 mg/dl (range 0.9–5.6 mg/dl). Of patients with available data, 33% (3 of 9) had hematuria, and 67% of patients had albuminuria (1 of 9) or subnephrotic range proteinuria (5 of 9). Half (5 of 10) of the patients had hypertension. Half (4 of 8) of the patients were nonsmokers, and half (4 of 8) were former smokers. All 10 patients had renal mass(es) and 40% (4 of 10) had extrarenal involvement based on symptoms and imaging studies (upper and lower respiratory tract, pituitary gland, lacrimal gland, retroperitoneum, leptomeninges, pancreas, and spleen). Antimyeloperoxidase or perinuclear (p)-ANCA and antiproteinase 3 or cytoplasmic (c)-ANCA was detectable in all 10 patients tested by commercially available enzyme-linked immunosorbent assay, multiplex flow immunoassay, or indirect immunofluorescence, as applicable. Antinuclear antibody was positive in 1 of 6 patients tested. Serum C3 and C4 complement levels were normal in all tested patients. Serum IgG4 was elevated in 67% (2 of 3) of tested patients and ranged from 82 mg/dl to 231 mg/dl (normal range: 2.4–121 mg/dl). Coexistent medical conditions included ascending aortic and pulmonary aneurysms (1 of 10), breast carcinoma (1 of 10), hypothyroidism (3 of 10), Gilbert’s syndrome (1 of 10), meningioma (1 of 10), and benign prostatic hyperplasia (1 of 10). None of the patients had systemic lupus erythematosus. One patient with panhypopituitarism, chronic sinusitis, lacrimal gland mass and retroperitoneal fibrosis was described clinically as having IgG4 pseudolymphoma syndrome despite having a positive ANCA. Follow-up data were available for 8 patients. Two of these patients had died; information about the cause of death was not available. Average follow-up for the patients was 118 months (range: 7−218 months); tested patients had follow-up mean serum creatinine of 2.39 mg/dl (range: 0.69−5.06 mg/dl). Information about treatment was not available for 3 of the patients. The remaining patients were treated with steroids and/or rituximab.

**Table 1:** Clinical characteristics, radiologic findings and treatment

Pt#	Age	Sex	S Alb (g/dl)	S Cr (mg/dl)	p-ANCA or anti-MPO pos	c-ANCA or anti-PR3 pos	UA	Radiology	Indication for biopsy	S Cr on last follow-up (mg/dl)	Duration of Follow-up (Mo)	Death	Treatment
1	68	M	3.9	1.4	y		Normal	Multiple bilateral round/round flat masses, low-intermediate density, mildly heterogeneous, cortical based 2–3 cm renal masses with mild bulge; pancreatic tail cystic lesion; ill-defined 2 cm hepatic lesion; left kidney 4.5 cm cyst	Renal mass	1.62	218	No	Methotrexate and prednisone
2	61	M	4	1.1	y		Normal	Right kidney 3.5 cm low density mass, heterogeneous appearance on ultrasonography; brain mass (biopsy -meningioma), prostate mass (biopsy-benign prostatic hyperplasia), lung nodules, sinus opacification and membrane thickening	Renal mass	0.9	207	No	Azathioprine and prednisone
3	55	M	NA	0.9	y		Normal	Two bilateral round/round-flat wedge shaped, intermediate density, mildly heterogeneous, cortical based renal masses with mild bulge; bilateral hilar and mediastinal lymphadenopathy, pleural thickening, lung nodules, retroperitoneal thickening, lacrimal gland mass, pituitary infundibular enlargement, inflammatory changes in paranasal sinuses	Renal mass	1.49	137	No	Rituximab and steroids
4	68	F	2.3	4.9	y		Microscopic hematuria and subnephrotic range proteinuria	Renal mass and multiple organ masses; pulmonary infiltrates; right pelvic cystic lesion, hepatic lesion	Renal failure; rule out metastasis	NA	NA	NA	NA
5	81	F	NA	1.9	y		No hematuria; subnephrotic range proteinuria	Multiple bilateral round/round/flat wedge shaped, intermediate density, moderately heterogeneous, cortical based renal masses with some scar	Renal mass	6.29	143	No	Prednisone
6	71	M	NA	5.6	y		No hematuria; subnephrotic range proteinuria	Right single 2.5 cm exophytic renal mass, mildly heterogeneous, low density, hypoechoic on ultrasonography	Acute renal failure and renal mass	2.3	11	Yes	Methylprednisolone, prednisone and rituximab
7	76	F	NA	5.07		y	Hematuria and subnephrotic range proteinuria	Right 2.6 cm renal mass; bilateral renal cysts	Renal mass	4.74	21	Yes	Prednisone
8	63	M	NA	4.35		y	Hematuria and trace albuminuria	Left renal mass; sinus opacification	Acute renal failure and renal mass	2.32	8	Yes	Methylprednisolone, prednisone and rituximab
9	68	M	NA	NA	y		NA	Left kidney mass	Renal mass	0.69	7	No	NA
10	63	F	4	1.03	y		No hematuria; subnephrotic range proteinuria	Bilateral 2–4.5 cm renal masses	Renal mass	NA	NA	NA	NA

S Alb, serum albumin; ANCA, antineutrophil cytoplasmic antibody; c-ANCA, cytoplasmic antineutrophil cytoplasmic antibody; F, female; M, male; MPO, myeloperoxidase; NA, not available; p-ANCA, perinuclear antineutrophil cytoplasmic antibody; Pos, positive; PR3, proteinase 3; Pt, patient; S Cr, serum creatinine; UA, urinalysis; y, yes.

### Radiological Findings

**Figure 1:**
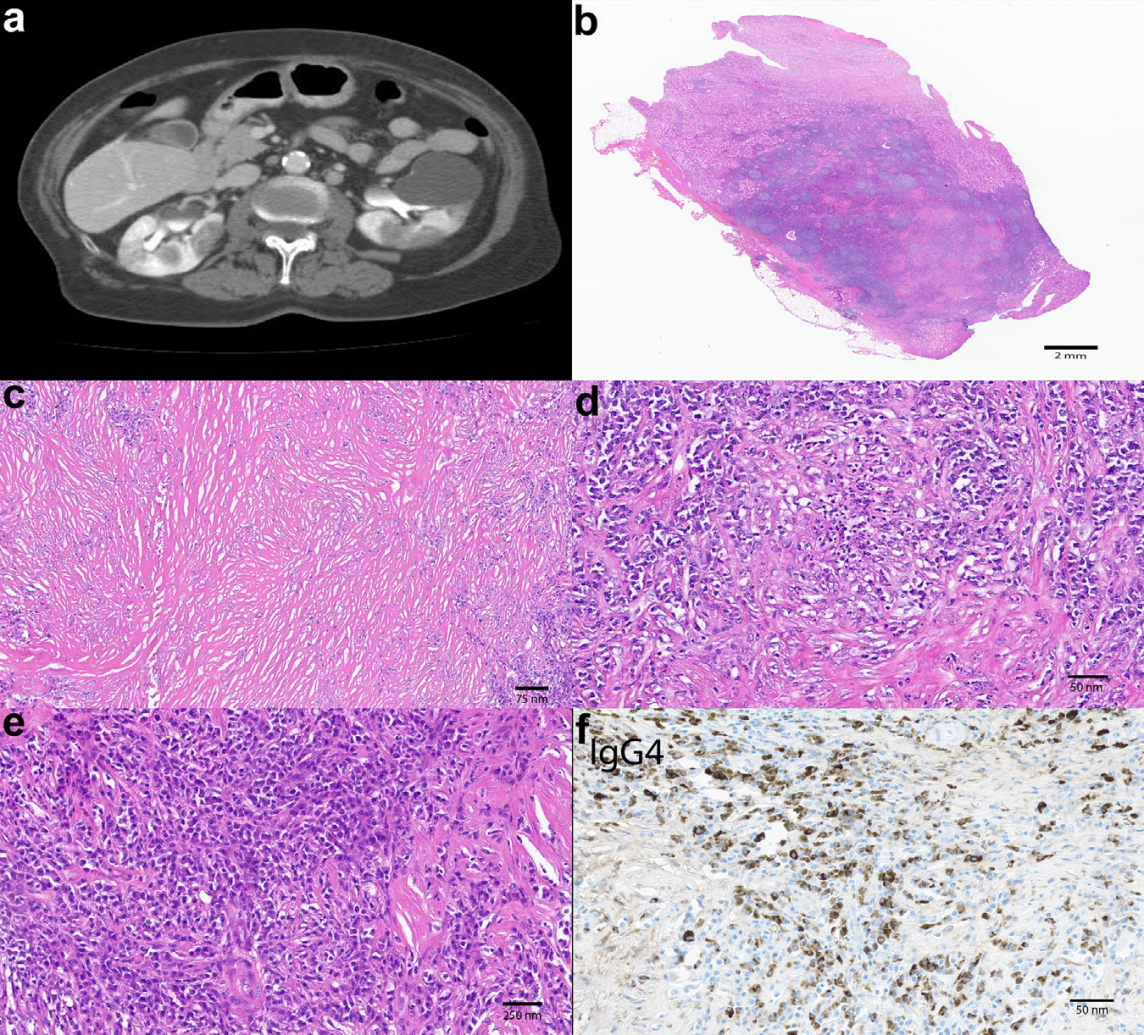
(a) Bilateral renal masses with low to intermediate density on computerized tomography (CT) imaging. (b) Renal parenchyma showing fibrosis and dense chronic inflammation and lymphoid follicles (Magnification: 0.3×). (c) Low power view shows storiform fibrosis and inflammation (magnification: 100×). (d) Intermediate power view showing focal karyorrhectic debris intermixed with interstitial inflammatory infiltrate (magnification: 200×). (e) Interstitial inflammatory infiltrate with predominant plasma cells (magnification: 250×). (f) Intermediate power view showing increased IgG4+ plasma cells in the interstitial inflammation (magnification: 200×).

All 10 patients had renal mass(es) that were predominantly cortical based, and heterogeneous on ultrasonography with low to intermediate density on computerized tomography (CT) imaging (see Figure 1). One of the renal masses was exophytic. Four of ten of the patients (40%) had multiple and bilateral renal masses.

Extrarenal radiographic findings included cystic lesion in pancreatic tail (1 of 10), hepatic lesions (2 of 10), bilateral hilar and mediastinal lymphadenopathy (1 of 10), pleural thickening (1 of 10), leptomeningeal thickening (1 of 10), pulmonary nodules or infiltrates (3 of 10), lacrimal gland enlargement (1 of 10), pituitary and infundibular enlargement (1 of 10), and sinus opacification or membrane thickening (3 of 10).

### Pathologic Characteristics

Seven patients had renal needle core biopsies, 1 had a renal wedge biopsy, and 2 underwent partial nephrectomy. The renal specimen findings are detailed in Supplementary Table S1. Glomerular sampling for light microscopy ranged from 3 to 105 glomeruli (mean 26). Cellular or fibrocellular crescents were present in 6 of 10 cases (60%); therefore, 40% of cases did not show cellular or fibrocellular crescents. Mean percentage of cellular or fibrocellular crescents and necrosis was 17% (range 0%−59%). All renal specimens had focal to diffuse interstitial inflammation comprising a mixed inflammatory infiltrate, including neutrophils, eosinophils, and plasma cells. Of all the cases, 7 of 9 (78%) had increased IgG4+ plasma cells (>10 IgG4+ plasma cells/40x high power microscopic field (hpf)); 5 of 9 (56%) had increased IgG4+/IgG+ plasma cell ratio (>0.4) (range: 0.05−0.67) (see Figure 1). Interstitial fibrosis and tubular atrophy were variable as follows: mild in 3 of 10 (30%), moderate in 1 of 10 (10%), and severe in 5 of 10 (50%). Of all cases, 4 of 10 (40%) showed granulomatous inflammation in the interstitium or sometimes surrounding glomeruli. Renal capsular and/or extracapsular extension of the inflammation was present in 2 of 4 cases. In the remaining cases, renal capsule was not sampled, precluding assessment of capsular extension. Interstitial karyorrhexis was seen in 4 of 10 (40%) of the cases. Necrotizing arteritis was present in 2 of 10 (20%) cases.

Immunofluorescence (IF) and electron microscopy (EM) were performed in 5 cases. IF was negative or showed a pauci-immune staining pattern in glomeruli; no cases showed tubular basement membrane deposits. EM showed an absence of tubular basement membrane immune deposits in all examined cases.

## Discussion

AAV is a systemic vasculitis associated with a positive serum ANCA that can involve multiple organs, including the retroperitoneum and rarely presents as unilateral or bilateral renal masses.S6 It is worth noting that testing for antimyeloperoxidase and antiproteinase 3 is more specific than c-ANCA testing and p-ANCA testing, because p-ANCA may be falsely positive in the setting of a positive antinuclear antibody (ANA). In renal biopsies, AAV generally shows a pauci-immune crescentic and necrotizing glomerulonephritis. The tubulointerstitial compartment can be involved by a mixed inflammatory infiltrate as well as fibrosis that may resemble a storiform pattern (see Figure 1). These histological findings pose a diagnostic challenge for pathologists because these features of AAV-associated renal masses overlap with those seen in IgG4-RD.

IgG4-RD is an immune mediated fibroinflammatory condition associated with increased serum IgG4 in most patients. It can involve multiple organs, including pancreas, salivary glands, lacrimal glands, kidney, and the retroperitoneum.S7 ANCA positivity is an exclusion criterion for IgG4-RD according to the 2019 The American College of Rheumatology/ European League Against Rheumatism (ACR/EULAR) classification criteria, based on characteristics of IgG4-RD cases and mimickers.S8 IgG4-RD is histologically characterized by a dense lymphoplasmacytic infiltrate and storiform fibrosis. Proposed histological diagnostic criteria for IgG4-RD includes dense lymphoplasmacytic infiltrate with increased IgG4+ plasma cells defined as >10 IgG4+ plasma cells/hpf and/or IgG4+/IgG+ plasma cell ratio of >40% or 0.4.S9

Retroperitoneal fibrosis may be a manifestation of IgG4-RD or AAV.S3,S10–S14 Of note, 1 patient with AAV in the current study had retroperitoneal fibrosis with normal serum IgG4 and positive p-ANCA and antimyeloperoxidase. This patient had a lacrimal gland mass biopsy that showed increased (>30/hpf) IgG4+ plasma cells.

Notably, 4 of 10 cases (40%) in our study did not show a necrotizing and crescentic glomerulonephritis; these cases in particular may be misdiagnosed as IgG4-RD. These cases showed focal to diffuse interstitial inflammation comprised of a mixed inflammatory infiltrate, including neutrophils, and IgG+ and IgG4+ plasma cells. The histological findings of dense lymphoplasmacytic infiltrates with increased IgG4+ plasma cells is reported in AAV, similar to IgG4-RD. Chang *et al.*S6 showed that out of 26 cases confirmed as AAV by clinical and pathological assessment, 8 cases (31% of total) had a marked increase in IgG4+ plasma cells (>30/hpf) and >0.4 IgG4+/IgG+ plasma cell ratio. Similarly, Kawano *et al.*S15 reported 6 AAV cases (6 of 10, 60% of total) that showed increased plasma cells (>30/hpf) in the interstitial inflammatory infiltrate and 4 cases (4 of 10, 40% of total) with >10 IgG4+ plasma cells/hpf, and >0.4 IgG4+/IgG+ plasma cell ratio. Raissian *et al.*S3 have also showed at least a moderate increase in interstitial IgG4+ plasma cells in 7 of 26 cases (27% of total) of pauci-immune glomerulonephritis biopsies. Similarly, in this study we found that the interstitial inflammatory infiltrate had increased IgG4+ plasma cells (>10/hpf) in 7 of 9 (78%) of cases and IgG4+/IgG+ plasma cell ratio was >0.40 in 44% (4 of 9) of cases.

In this study, histological findings of focal interstitial karyorrhexis, necrosis, or neutrophilic infiltrates were identified in all AAV cases with or without crescentic and necrotizing glomerulonephritis. These histological findings are helpful in determining renal masses to be associated with AAV despite having increased IgG4+ plasma cells in the interstitial inflammatory infiltrate and a background of “storiform” fibrosis (see Figure 1). Granulomas may be present in masses in kidneys or other organs; the presence of granulomas essentially excludes a diagnosis of IgG4-RD. Of our cases, 4 of 10 (40%) had granulomatous inflammation; 3 of these cases were associated with increased IgG4+ plasma cells (>30/hpf) and increased IgG4+/IgG+ plasma cell ratio (>0.4). Therefore, masses suspected to represent IgG4-RD based on histological findings of dense lymphoplasmacytic infiltrates with increased IgG4+ plasma cells and increased IgG4+/IgG+ plasma cell ratio, and storiform fibrosis need careful examination for interstitial karyorrhexis, necrosis, neutrophilic infiltrates, or granulomatous inflammation. These histological findings favor a mass to be AAV. Renal capsular extension of inflammation was seen in a subset of our AAV cases; this feature has also been described in IgG4-TIN and so it does not distinguish these entities.S16 Supplemental Table S2 summarizes the clinical and histomorphological findings in AAV and IgG4-RD.

The clinical significance of distinguishing between AAV and IgG4-RD lies in the differences in prognosis and management strategies. AAV and IgG4-RD are both treated with corticosteroids and rituximab, however there is difference in the drug schedules.S17–S19 Renal masses associated with increased IgG4+ plasma cells can also be seen in other conditions, including chronic pyelonephritis, xanthogranulomatous pyelonephritis, and Erdheim-Chester syndrome (Supplementary Table S3).

In conclusion, we identified histological findings that can distinguish AAV associated renal masses from those seen in IgG4-RD. Inflammatory infiltrates with elevated IgG4+ plasma cells and elevated IgG4+/IgG+ plasma cell ratio in a background of fibrosis should raise the differential diagnoses of mass-forming AAV and IgG4-RD. Interstitial karyorrhexis/necrosis, neutrophils, and glomerulonephritis favor a diagnosis of AAV despite dense lymphoplasmacytic infiltrates with increased IgG4+ plasma cells and storiform fibrosis. The distinction between AAV and IgG4-RD is important due to differences in prognosis and management. It is also important for pathologists to remember that renal masses with increased plasma cells can be seen in other conditions. These causes can be distinguished based on clinical and additional histopathologic findings in conjunction with appropriate laboratory testing.

## Disclosure

All the authors declared no competing interests.

## Supplementary Material

Supplementary File (PDF)

**Supplementary References.**

**Table S1.** Histological findings on LM, IF and EM

**Table S2.** Comparison of ANCA associated vasculitis and IgG4-related disease

**Table S3.** Renal masses with increased IgG4+ plasma cells
