# Supplementary material for: Clinicopathologic Findings in Mass Forming ANCA-Associated Vasculitis
Source: Kidney Int Rep. 2022 Sep 29;7(12):2709–13. doi: 10.1016/j.ekir.2022.09.019 (PMC9727509; doi:10.1016/j.ekir.2022.09.019)
Supplement: Supplementary File (PDF) [file mmc1.pdf]

Supplementary Materials

Methods

A review of native renal biopsies and nephrectomy specimens evaluated at Mayo Clinic, Rochester, identified 12 patients with AAV-associated masses involving the kidney or other organs. Among patients with a kidney histologic specimen, inclusion criteria for the study were as follows: (1) inflammatory mass(es) in the kidney or other organs; and (2) positive serum ANCA (anti-MPO, anti-PR3 or c- or p-ANCA), pauci-immune necrotizing and crescentic glomerulonephritis, or both. Standard processing of renal biopsies included light microscopy (LM), immunofluorescence (IF), and electron microscopy (EM). 5 cases did not have IF and EM performed. For LM, all cases were stained with hematoxylin and eosin, periodic acid-Schiff, Masson's trichrome, and Jones methenamine silver. For IF, 4-micron cryostat sections were stained with polyclonal fluorescein isothiocyanate–conjugated antibodies to IgG, IgM, IgA, C3, C1q, kappa, lambda, fibrinogen, and albumin (Dako, Carpinteria, CA). Immunohistochemical staining for IgG4 and IgG were performed on all the cases using the DAKO dual multimer system or DAKO advance 2 stops multimer system (DAKO, Carpinteria, CA). Monoclonal IgG4 antibody (clone HP6025; Zymed, San Francisco, CA) and polyclonal rabbit antihuman IgG antibody (DAKO) were used for the study. IgG4+ and IgG+ plasma cells were counted using method previously described by Chang et al[S6]. An increase in IgG4+ plasma cells was defined as IgG4+ plasma cells > 10/ 40x microscopic field (hpf) in the most concentrated area, and the IgG4+/IgG+ plasma cell ratio of > 0.4 was considered increased.

The radiologic characteristics of renal masses were reviewed by a radiologist for available ultrasonography and computed tomography images.

Supplementary References

S1. Nagashima T, Maguchi S, Terayama Y, et al. P-ANCA-positive Wegener's granulomatosis presenting with hypertrophic pachymeningitis and multiple cranial neuropathies: case report and review of literature. *Neuropathology*. Mar 2000;20(1):23-30. doi:10.1046/j.1440-1789.2000.00282.x

S2. Yamamoto T, Tkahata K, Kamei S, Ishikawa M, Matsumoto D, Suzuki K. Granulomatosis with polyangiitis presenting as a solitary renal mass: A case report with imaging and literature review. *Radiol Case Rep*. Mar 2021;16(3):736-741. doi:10.1016/j.radcr.2021.01.014

S3. Raissian Y, Nasr SH, Larsen CP, et al. Diagnosis of IgG4-related tubulointerstitial nephritis. *J Am Soc Nephrol*. Jul 2011;22(7):1343-52. doi:10.1681/ASN.2011010062

S4. Chang SY, Keogh K, Lewis JE, Ryu JH, Yi ES. Increased IgG4-Positive Plasma Cells in Granulomatosis with Polyangiitis: A Diagnostic Pitfall of IgG4-Related Disease. *Int J Rheumatol*. 2012;2012:121702. doi:10.1155/2012/121702

S5. Danlos FX, Rossi GM, Blockmans D, et al. Antineutrophil cytoplasmic antibody-associated vasculitides and IgG4-related disease: A new overlap syndrome. *Autoimmun Rev*. Oct 2017;16(10):1036-1043. doi:10.1016/j.autrev.2017.07.020

S6. Chang SY, Keogh KA, Lewis JE, et al. IgG4-positive plasma cells in granulomatosis with polyangiitis (Wegener's): a clinicopathologic and immunohistochemical study on 43 granulomatosis with polyangiitis and 20 control cases. *Hum Pathol*. Nov 2013;44(11):2432-7. doi:10.1016/j.humpath.2013.05.023

S7. Villa-Forte A, Hoffman GS. Wegener's granulomatosis presenting with a renal mass. *J Rheumatol*. Feb 1999;26(2):457-8.

S8. Wallace ZS, Zhang Y, Perugino CA, et al. Clinical phenotypes of IgG4-related disease: an analysis of two international cross-sectional cohorts. *Ann Rheum Dis*. Mar 2019;78(3):406-412. doi:10.1136/annrheumdis-2018-214603

S9. Wallace ZS, Naden RP, Chari S, et al. The 2019 American College of Rheumatology/European League Against Rheumatism classification criteria for IgG4-related disease. *Ann Rheum Dis*. Jan 2020;79(1):77-87. doi:10.1136/annrheumdis-2019-216561

S10. Vaglio A, Salvarani C, Buzio C. Retroperitoneal fibrosis. *Lancet*. Jan 21 2006;367(9506):241-51. doi:10.1016/S0140-6736(06)68035-5

S11. Lian L, Wang C, Tian JL. IgG4-related retroperitoneal fibrosis: a newly characterized disease. *Int J Rheum Dis*. Nov 2016;19(11):1049-1055. doi:10.1111/1756-185X.12863

S12. Fujimori N, Ito T, Igarashi H, et al. Retroperitoneal fibrosis associated with immunoglobulin G4-related disease. *World J Gastroenterol*. Jan 7 2013;19(1):35-41. doi:10.3748/wjg.v19.i1.35

S13. Mizushima I, Kawano M. Renal Involvement in Retroperitoneal Fibrosis: Prevalence, Impact and Management Challenges. *Int J Nephrol Renovasc Dis*. 2021;14:279-289. doi:10.2147/IJNRD.S239160

S14. Akiyama M, Kaneko Y, Takeuchi T. Characteristics and prognosis of ANCA-positive retroperitoneal fibrosis: A systematic literature review. *Autoimmun Rev*. Oct 2020;19(10):102642. doi:10.1016/j.autrev.2020.102642

S15. Kawano M, Mizushima I, Yamaguchi Y, et al. Immunohistochemical Characteristics of IgG4-Related Tubulointerstitial Nephritis: Detailed Analysis of 20 Japanese Cases. *Int J Rheumatol*. 2012;2012:609795. doi:10.1155/2012/609795

S16. Yoshita K, Kawano M, Mizushima I, et al. Light-microscopic characteristics of IgG4-related tubulointerstitial nephritis: distinction from non-IgG4-related tubulointerstitial nephritis. *Nephrol Dial Transplant*. Jul 2012;27(7):2755-61. doi:10.1093/ndt/gfr761

S17. Brito-Zeron P, Kostov B, Bosch X, Acar-Denizli N, Ramos-Casals M, Stone JH. Therapeutic approach to IgG4-related disease: A systematic review. *Medicine (Baltimore)*. Jun 2016;95(26):e4002. doi:10.1097/MD.0000000000004002

S18. Stone JH, Merkel PA, Spiera R, et al. Rituximab versus cyclophosphamide for ANCA-associated vasculitis. *N Engl J Med*. Jul 15 2010;363(3):221-32. doi:10.1056/NEJMoa0909905

S19. Carruthers MN, Topazian MD, Khosroshahi A, et al. Rituximab for IgG4-related disease: a prospective, open-label trial. *Ann Rheum Dis*. Jun 2015;74(6):1171-7. doi:10.1136/annrheumdis-2014-206605

Supplemental Table S1: Histological findings on LM, IF and EM

Pt#	%global GS	% cellular/fibrocellular crescent	IgG4+ plasma cells/hpf	IgG4+/IgG+ plasma cell ratio	Interstitial granulomatous inflammation	IF	EM
1	11	21	>30	0.23	Yes	NA	NA
2	1	59	>30	0.60	Yes	NA	NA
3	0	0	11-30	0.14	No	NA	NA
4	33	25	<10	0.44	Yes	Crescents with fibrinogen	Negative
5	100	0	>30	0.38	No	Interstitial granular staining within areas of fibrosis for IgA, IgG, IgM, C1q, C3, albumin, fibrinogen, kappa, lambda	No TBM or interstitial deposits
6	12	28	>30	0.67	No	Crescents with fibrinogen and interstitial staining with fibrinogen	No TBM deposits
7	74	24	<5	0.06	No	Focal staining with fibrinogen	Negative
8	0	10	NA	NA	No	Negative	Negative
9	0	0	>30	0.40	Yes	NA	NA
10	80	80	>50	0.4	No	NA	NA

LM: Light microscopy, IF: Immunoflorescence, EM: Electron Microscopy, NA: Not available

Supplemental Table S2: Comparison of ANCA associated vasculitis and IgG4-related disease

	ANCA associated vasculitis	IgG4-RD
Clinical Presentation	Rapidly progressive glomerulonephritis, renal mass(es)	Acute or chronic renal failure, renal mass(es)
Commonly involved extrarenal tissues	Lungs, nasal sinuses, retroperitoneum/periureteral, multiple sites (microscopic polyangiitis)	Submandibular glands, lymph nodes, orbit, pancreas, retroperitoneum, lung, parotid gland, aorta, bile duct
Glomerulonephritis (GN)	Pauci-immune necrotizing/crescentic GN	Membranous GN, mild mesangial immune-complex GN; Glomerular "pseudocrescents" may accompany IgG4-TIN*
Tubulointerstitial nephritis	Yes, plasma cell rich	Yes, plasma cell rich
**Increased IgG4+ plasma cells or IgG4/IgG+ plasma cell ratio**	**Yes**	**Yes**
**Granulomatous inflammation**	**Yes**	**No**
**Interstitial karyorrhexis or necrosis**	**Yes**	**No**
**Neutrophils**	**Yes**	**Few, focal**
Arteritis	Yes, necrotizing	Yes (rare), non-necrotizing
**Plasma cell rich arteritis**	**No**	**Yes**
Treatment	Steroids, cyclophosphamide, rituximab	Steroids, rituximab

***"Pseudocrescents" refers to a lesion in which the tubulointerstitial fibroinflammatory process involves Bowman's capsule in IgG4-TIN; this does not represent a true glomerulonephritis.**

Supplemental Table S3: Renal masses with increased IgG4+ plasma cells

Diagnosis	Etiology/Cause	Histological features
Chronic pyelonephritis	Chronic renal damage from persistent or recurrent bacterial infection	The tubulointerstitial inflammation is predominantly composed of lymphocytes, monocytes and plasma cells, which may include IgG4+ plasma cells. There is associated scarring of renal parenchyma that can involve the renal pelvis and calyces.
Xanthogranulomatous pyelonephritis	In setting of nephrolithiasis or obstructive uropathy	Diffuse or focal renal parenchymal involvement by granulomatous inflammation with abundant foamy histiocytes admixed with mixed inflammatory infiltrate (neutrophils, plasma cells and lymphocytes). There can be an associated variable degree of renal tubular atrophy, lymphoid aggregates with germinal centers, and marked fibroblastic proliferation.
Erdheim-Chester disease (ESD)	Non-Langerhans cell histiocytic disorder	Sheets of foamy histiocytes and an inflammatory infiltrate of plasma cells.
